# Application of immune checkpoint inhibitors for resectable gastric/gastroesophageal cancer

**DOI:** 10.3389/fphar.2024.1391562

**Published:** 2024-05-09

**Authors:** Feizhi Lin, Yongming Chen, Bowen Huang, Shenghang Ruan, Jun Lin, Zewei Chen, Chunyu Huang, Baiwei Zhao

**Affiliations:** State Key Laboratory of Oncology in South China, Guangdong Provincial Clinical Research Center for Cancer, Sun Yat-Sen University Cancer Center, Guangzhou, China

**Keywords:** perioperative treatment, gastric cancer, immunotherapy, neoadjuvant therapy, adjuvant therapy, chemotherapy

## Abstract

Gastric/gastroesophageal junction (G/GEJ) cancer represents a significant global health challenge. Radical surgery remains the cornerstone of treatment for resectable G/GEJ cancer. Supported by robust evidence from multiple clinical studies, therapeutic approaches, including adjuvant chemotherapy or chemoradiation, and perioperative chemotherapy, are generally recommended to reduce the risk of recurrence and enhance long-term survival outcomes post-surgery. In recent years, immune checkpoint inhibitors (ICIs) have altered the landscape of systemic treatment for advanced or metastatic G/GEJ cancer, becoming the standard first-line therapy for specific patients. Consequently, exploring the efficacy of ICIs in the adjuvant or neoadjuvant setting for resectable G/GEJ cancer is worthwhile. This review summarizes the current advances in the application of ICIs for resectable G/GEJ cancer.

## Introduction

Gastric/gastroesophageal junction (G/GEJ) cancer represents a significant global cancer burden. In 2020, G/GEJ cancer accounted for over one million new cases and roughly 769,000 deaths, ranking as the fifth most frequently diagnosed cancer and the fourth leading cause of cancer-related mortality (Siegel et al., 2021; Sung et al., 2021). There is a geographical variation in incidence rates, with Eastern Asia and Eastern Europe reporting the highest, while Northern America, Northern Europe, and African regions report relatively low rates (Sung et al., 2021). Notably, an upward trend in the incidence among young adults is observed globally, transcending traditional high- and low-risk regions (Sung et al., 2021). Owing to the subtle symptoms of early-stage G/GEJ cancer, a considerable proportion of patients receive diagnoses at advanced stages, resulting in a poor prognosis (Guan et al., 2023).

Distal, subtotal or total gastrectomy along with D2 lymphadenectomy is defined as standard surgery for resectable G/GEJ cancer (Wang et al., 2021; Ajani et al., 2022; Lordick et al., 2022). Various clinical trials have confirmed the superiority of adjuvant and neoadjuvant-adjuvant therapy compared to surgery alone for resectable cases (Guan et al., 2023). In East Asian countries like Japan and Korea, where screening program is widely conducted, treatment typically involves D2 gastrectomy followed by adjuvant chemotherapy, even though neoadjuvant therapy is becoming more common. Conversely, western countries put more focus on neoadjuvant-adjuvant therapy in combination with surgery (Suh and Yang, 2015; Allemani et al., 2018; Yanagimoto et al., 2023). Despite the significant progress in the therapeutic strategies and surgical techniques, there remains a rather high risk of recurrence and metastasis in resectable cases, with the 5-year survival rates showing a substantial decline in patients beyond stage II (Li et al., 2018).

**FIGURE 1:**
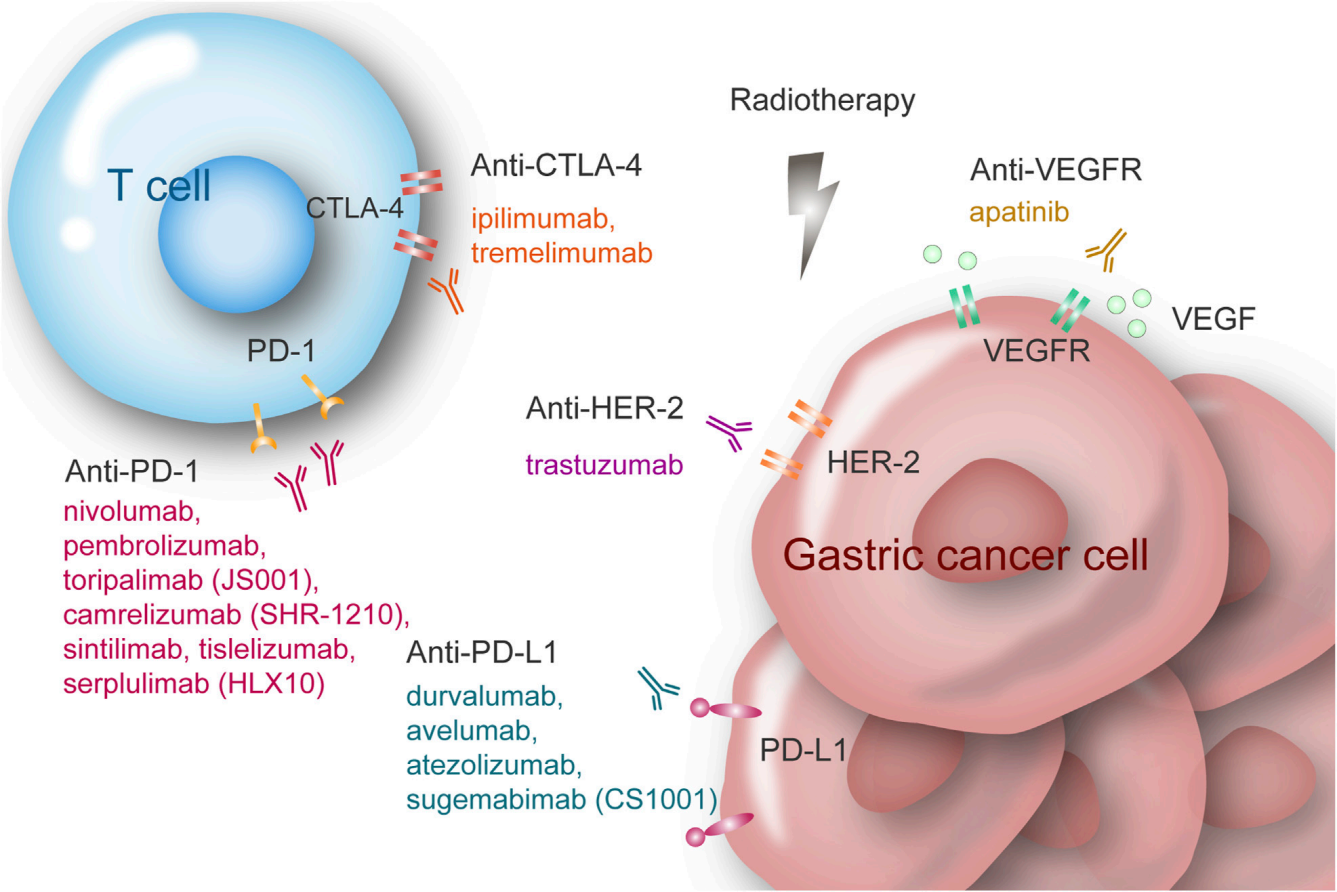
Multiple modalities for locally advanced gastric/gastroesophageal cancer.

The emergence of immune checkpoint inhibitors (ICIs) has revolutionized the management of various solid malignancies. Key inhibitory immune checkpoints, including cytotoxic T-lymphocyte-associated protein 4 (CTLA-4 or CD152), programmed cell death protein 1 (PD-1 or CD279), and programmed death-ligand 1 (PD-L1 or CD274), serve as crucial modulators of the immune system by downregulating T cell activity. Cancer cells exploit this mechanism to evade immune detection, often leading to worse outcomes (Bagchi et al., 2021). Immune checkpoint inhibitors (ICIs), specifically designed monoclonal antibodies, counteract these checkpoints, potentiating T cell-mediated tumor destruction (Marin-Acevedo et al., 2021). Common ICIs are categorized based on their specific targets: anti-PD-1 antibodies (e.g., nivolumab and pembrolizumab), anti-PD-L1 antibodies (e.g., durvalumab and avelumab), and anti-CTLA-4 antibodies (e.g., ipilimumab and tremelimumab) (Figure 1).

For advanced G/GEJ cancer, ATTRACTION-2 validated the efficacy of ICIs in the later-line treatment (Kang et al., 2017). Furthermore, results from CHECKMATE-649, ORIENT-16 indicated that the combination of ICIs and chemotherapy offers survival benefits as a first-line treatment (Janjigian et al., 2021a; Xu et al., 2021). Immunotherapy has become the standard first-line treatment for advanced G/GEJ cancer patients with PD-L1 CPS ≥5 (4–6). Given this, various clinical trials are investigating its efficacy in the postoperative and perioperative setting in resectable cases (Guan et al., 2023). Current research is focused on exploring the synergetic effects of combining immunotherapy with other modalities such as chemotherapy, radiotherapy, and targeted therapy (Figure 1). In this review, we summarize the clinical trials concerning the application of ICIs in resectable G/GEJ cancer.

## Application of ICIS in the adjuvant setting

Surgery alone for locally advanced GC yields unsatisfied outcomes, with a postoperative 5-year overall survival (OS) rate below 50% even after D2 gastrectomy (Smith et al., 2005). Adjuvant therapy aims to reduce microscopic disease and prevent recurrence. Stage II or III cancer patients undergoing radical surgery are advised to receive adjuvant chemotherapy, especially in Asian populations, as supported by multiple clinical trials. The Japanese ACTS-GC trial showed that adjuvant S-1 monotherapy for 1 year after surgery provides a survival advantage over surgery alone in stage II/III GC patients (Sasako et al., 2011). The CLASSIC trial, conducted in South Korea, China, and Taiwan, confirmed the advantages of adjuvant capecitabine and oxaliplatin (CAPOX) for stage II-IIIB G/GEJ cancer patients undergoing D2 gastrectomy *versus* surgery only (Bang et al., 2012). The Korean ARTIST two trial compared surgery followed by 1 year of S-1 monotherapy, 6 months of S-1 combined with oxaliplatin (SOX), or a combination of radiotherapy plus SOX (SOXRT) for lymph node-positive stage II/III GC patients. The study revealed that adjuvant SOX extended disease-free survival (DFS) compared to S-1 alone (Park et al., 2021). In the Chinese RESOLVE trial, patients with cT4aN + or cT4bNany G/GEJ cancer were compared across three treatment arms: surgery followed by adjuvant CAPOX, adjuvant SOX, or perioperative SOX. Result showed that adjuvant SOX was noninferior to adjuvant CAPOX in patients with cT4aN + or cT4bNany G/EGJ cancer in DFS(20). The JACCRO GC-07 trial in Japan verified the superiority of docetaxel plus S-1 (DS) over S-1 monotherapy as postoperative treatment in pathological stage III GC patients (Yoshida et al., 2019; Kakeji et al., 2022). These findings provide insights into the selection of adjuvant therapies for stage II-III gastric cancer. S-1 alone may be preferred for patients with stage II cancer or those with a poor performance status, while combination therapies like CAPOX, SOX, or DS are recommended for patients with pathological stage III disease (Wang et al., 2021; Japanese Gastric Cancer Association, 2023). The integration of ICIs into adjuvant therapy for operable G/GEJ cancer is currently being investigated to assess potential survival benefits (Table 1).

**TABLE 1:** Clinical trials of ICIs in adjuvant setting.

Trial	Patients	Agent (target)	Phase, arm	Study design	Size	Primary endpoint	
ATTRACTION-5 (NCT03006705)	-	Nivolumab (PD-1)	III, two-arm	Nivolumab + S-1/CAPOX	377	3-year RFS	68.4%
				Placebo + S-1/CAPOX	378		65.3%
JUPITER-15 (NCT05180734)	-	Toripalimab (PD-1)	III, two-arm	Toripalimab + SOX/XELOX	340	DFS	
				Placebo + SOX/XELOX	340		
NCT05184946	-	Camrelizumab (PD-1)	II, two-arm	Camrelizumab + SOX	36	3-year DFS	
				SOX only	36		
NCT05468138	dMMR/MSI-H	Sintilimab/Nivolumab (PD-1)	II/III, three-arm	Sintilimab/Nivolumab only	141	3-year DFS	
				SOX/XELOX only			
				observation			
NCT05769725	PD-L1CPS≥5/EBV+/dMMR/MSI-H	Serplulimab (PD-1)	II, two-arm	Serplulimab + DS	35	1-year DFS	
				DS only	35		
NCT04997837	D2/R0 resected with pN3	multiple PD-1 inhibitors	III, two-arm	PD-1+CRT	433	3-year DFS	
				Chemotherapy			

CAPOX: Capecitabine + Oxaliplatin; XELOX: Xeloda + Oxaliplatin; SOX: S-1 + Oxaliplatin; DS: Docetaxel + S-1; CRT: chemoradiotherapy; RFS: recurrence free survival; DFS: disease free survival.

The ATTRACTION-5 (NCT03006705) trial, presented at ASCO 2023, was a phase 3, Asian, double-blind, randomized study to evaluate the efficacy of nivolumab combined with adjuvant chemotherapy (either S-1 monotherapy or CAPOX) in patients with pathological stage III G/GEJ cancer who had undergone D2 (or more extensive) gastrectomy. 755 patients were randomly assigned to the Nivolumab plus chemotherapy (N + C) arm and the placebo plus chemotherapy (P + C) arm. The primary endpoint, centrally-assessed relapse-free survival (RFS), was not met (HR 0.90; 95% CI 0.69–1.18; *p* = 0.4363). The centrally-assessed 3-year RFS rates were 68.4% (95% CI 63.0–73.2) in the N + C group and 65.3% (95% CI 59.9–70.2) in the P + C group. Incidences of grade≥3 treatment-related adverse events (TRAEs), serious TRAEs, and TRAEs leading to treatment discontinuation were 54.4%, 25.3%, and 9.2% in the N + C group *versus* 46.8%, 10.7%, and 3.5% in the P + C group, respectively. Subgroup analysis showed that patients with PD-L1 expression ≥1% might benefit from N + C treatment. A majority of patients had low PD-L1 expression in the study cohorts may represent an important factor for the negative outcomes (Terashima et al., 2023). The subgroup analysis from CheckMate-649, along with a meta-analysis, have consistently found that in patients with low PD-L1 combined positive score (CPS), the therapeutic benefits of ICIs combined with chemotherapy may diminish (Janjigian et al., 2021a; Shitara et al., 2022; Yoon et al., 2022). This observation could be particularly relevant in the postoperative adjuvant setting, where significant changes in the tumor microenvironment (TME), including the near elimination of PD-L1 positive tumor cells, might impede the ability of ICIs to activate the immune system effectively.

JUPITER-15 (NCT05180734) is an ongoing, phase 3, global, double-blind study assessing the efficacy and safety of combining toripalimab with adjuvant chemotherapy (XELOX or SOX) in comparison to placebo with adjuvant chemotherapy. It includes patients who have undergone radical gastrectomy (R0, D2 or higher lymphadenectomy) and have a postoperative pathological stage II or III G/GEJ adenocarcinoma, regardless of PD-L1 expression. Another ongoing phase 2 randomized study NCT05184946 is exploring the efficacy and safety of camrelizumab plus SOX for adjuvant therapy of pathologic stage III G/GEJ adenocarcinoma compared to the standard SOX regimen. Given their similarity to the ATTRACTION-5 trial in design, these trials may not yield positive results.

According to a meta-analysis of MAGIC, CLASSIC, ARTIST, and ITACA-S, resectable mismatch repair deficiency (dMMR) or microsatellite instability-high (MSI-H) GC patients tend to have better outcomes with surgery alone than with postoperative adjuvant chemotherapy (Pietrantonio et al., 2019). However, these patients respond well to immunotherapy (Kang and Chau, 2020). The ongoing, phase 2, three-arm, randomized NCT05468138 trial aims to demonstrate that dMMR/MSI-H G/GEJ cancer patients who receive sintilimab or nivolumab monotherapy after D2 radical gastrectomy will have a more favorable prognosis than those receiving standard postoperative adjuvant chemotherapy (SOX/XELOX) or undergoing follow-up observation. Additionally, the phase 2 NCT05769725 trial is evaluating serplulimab in combination with DS *versus* DS alone as adjuvant treatment therapy in pathologic stage IIIc GC with PD-L1 CPS≥5/EBV+ (Epstein-Barr virus positive)/dMMR/MSI-H. These investigations will contribute to elucidating whether adjuvant immunotherapy can benefit specific patient populations.

Based on the results of ARTIST 2, adjuvant radiotherapy is not routinely recommended after D2 gastrectomy for GC due to its limited impact on reducing recurrence rates when added to SOX (19). However, in clinical practice, patients with advanced postoperative staging and a high risk of local recurrence (defined as inadequate safety margins, vascular tumor emboli, perineural invasion, advanced N-stage, or a high lymph node metastasis ratio) may consider adjuvant radiotherapy after comprehensive systemic treatment (Wang et al., 2021). NCT04997837 is a multicenter, randomized controlled, phase 3 study designed to assess the efficacy and safety of postoperative adjuvant chemotherapy CAPOX/SOX/FOLFOX (folinic acid, fluorouracil and oxaliplatin) with PD-1 inhibitors (nivolumab/toripalimab/pembrolizumab/tilelizumab/sintilimab/carrelizumab) and chemoradiotherapy (CRT) in comparison with adjuvant chemotherapy alone for patients with D2/R0 resected pN3 G/GEJ adenocarcinoma. Patients in the PD-1+CRT cohort will receive PD-1 inhibitors and chemotherapy for 6 weeks, followed by concurrent chemoradiotherapy (cCRT), another 6 weeks of PD-1 inhibitors and chemotherapy, and maintenance PD-1 inhibitors for up to 1 year after radiotherapy. While patients in the CT cohort will receive chemotherapy only for 6 months. The study aims to assess the 3-year DFS (primary endpoint), OS, RFS and adverse effects to identify the most effective treatment approach.

## Application of ICIS in the perioperative setting

Perioperative (neoadjuvant and adjuvant) therapy is a standard of care for resectable G/GEJ cancer. Conventional neoadjuvant chemotherapy and radiotherapy aims to reduce tumor size and improve surgical resectability, while neoadjuvant immunotherapy can boost tumor-specific T cells to enhance both intratumoral and systemic anti-tumor immunity (Lin et al., 2023; Topalian et al., 2023).

### ICIs in combination with chemotherapy

The MAGIC trial established the survival benefit of perioperative ECF (epirubicin, cisplatin, and fluorouracil) regimen plus surgery *versus* surgery alone in patients with operable G/GEJ, or lower esophageal adenocarcinoma (Cunningham et al., 2006). The French FNCLCC/FFCD trial revealed the similar efficacy of FP regimen (fluorouracil and cisplatin) to ECF (Ychou et al., 2011). The FLOT4-AIO trial has led to the replacement of ECF with the FLOT regimen (fluorouracil, leucovorin, oxaliplatin, and docetaxel) as the favored perioperative treatment in Europe (Al-Batran et al., 2019). The Korean PRODIGY trial showed that neoadjuvant DOS (docetaxel, oxaliplatin, and S-1) followed by surgery and adjuvant S-1 is superior to surgery plus adjuvant S-1 for resectable GC, despite some criticism regarding the postoperative S-1 monotherapy (Tougeron et al., 2022). In the Chinese RESOLVE trial, perioperative SOX therapy showed an improvement over adjuvant CAPOX therapy (Zhang et al., 2021). Based on these evidences, the treatment of neoadjuvant therapy followed by surgery and adjuvant therapy is recommended for resectable locally advanced GC in different countries (Wang et al., 2021; Ajani et al., 2022; Lordick et al., 2022). The FLOT regimen is most frequently used in western countries, while SOX is preferred in China. Ongoing research, including phase II and III trials, is investigating whether adding ICIs to perioperative treatment can improve survival outcomes for resectable G/GEJ cancer patients (Table 2).

**TABLE 2:** Clinical trials of ICIs plus chemotherapy in perioperative setting.

Trial	Patients	Agent (target)	Phase, arm	Study design	Size	pCR (%)	MPR (%)
NCT03939962	-	Camrelizumab (PD-1)	II, single-arm	Camrelizumab + FOLFOX	16	7.7	30.8
PANDA (NCT03448835)	-	Atezolizumab (PD-L1)	II, single-arm	Atezolizumab + DOC	20	45.0	70.0
NCT03488667	-	Pembrolizumab (PD-1)	II, single-arm	Pembrolizumab + mFOLFOX	35	19.2	-
DANTE (NCT03421288)	MSI-H,PD-L1 CPS≥1, TMB10/MB, or EBV+	Atezolizumab (PD-L1)	II/III, two-arm	Atezolizumab + FLOT	146	24	-
				FLOT only	149	15	-
ICONIC (NCT03399071)	-	Avelumab (PD-L1)	II, single-arm	Avelumab + FLOT	34	15	-
NCT04250948	-	Toripalimab (PD-1)	II, two-arm	Toripalimab + SOX/XELOX	54	24.1	44.4
				SOX/XELOX only	54	9.3	20.4
NCT04908566	-	Toripalimab (PD-1)	II, two-arm	Toripalimab + FOLFIRINOX	21	15.4	31.6
				Toripalimab + SOX	33	10.5	23.1
KEYNOTE-585 (NCT03221426)	-	Pembrolizumab (PD-1)	III, two-arm	Pembrolizumab + XP/FP/FLOT	502	13.0	-
				Placebo + XP/FP/FLOT	505	2.4	-
MATTERHORN (NCT04592913)	-	Durvalumab (PD-L1)	III, two-arm	Durvalumab + FLOT	474	19	27
				Placebo + FLOT	474	7	14
NCT04139135	PD-L1 CPS≥5	Serplulimab (PD-1)	III, two-arm	Serplulimab + SOX	321	-	-
				Placebo + SOX	321	-	-
NICE (NCT04744649)	PD-L1 CPS≥5 or dMMR/MSI-H, EBV+	Toripalimab (PD-1)	II, two-arm	Toripalimab + SOX/XELOX	55	-	-
				SOX/XELOX only	55	-	-
NCT04367025	PD-L1 CPS≥1	Camrelizumab (PD-1)	II, single-arm	Camrelizumab + SOX	70	-	-
MONEO (NCT03979131)	-	Avelumab (PD-L1)	II, single-arm	Avelumab + FLOT	40	-	-
NCT05101616	-	Camrelizumab (PD-1)	I/II, two-arm	Camrelizumab + nab-PTX + SOX	50	-	-
				nab-PTX + S-1+ oxaliplatin	50	-	-
TACTIC (NCT05593458)	-	Sintilimab (PD-1)	II, two-arm	Artery-infused oxaliplatin + S-1+ Sintilimab	95	-	-
				SOX + Sintilimab	95	-	-
NCT05994456	dMMR/MSI-H	Toripalimab (PD-1)	II, single-arm	Toripalimab only	24	-	-
NCT05836584	dMMR/MSI-H	Atezolizumab (PD-L1)	II, two-arm	Atezolizumab only	120	-	-
				Atezolizumab + FLOT/mFOLFOX/CAPOX	120	-	-

FOLFOX: Folinic acid + Fluorouracil + Oxaliplatin; mFOLFOX: modified FOLFOX; DOC: Docetaxel + Oxaliplatin + S-1; FLOT: Fluorouracil + Leucovorin + Oxaliplatin + Docetaxel; SOX: S-1 + Oxaliplatin; XELOX: Xeloda + Oxaliplatin; CAPOX: Capecitabine + Oxaliplatin; FOLFIRINOX: Fluorouracil + Leucovorin + Oxaliplatin + Irinotecan; XP: Xeloda + Cisplatin; FP: Fluorouracil + Cisplatin; nab-PTX: nab-paclitaxel; pCR: pathologic Complete Response; MPR: major pathologic response.

The result of the single-arm, phase 2 NCT03939962 study was reported at ASCO 2020. All 16 patients with resectable G/GEJ adenocarcinoma completed neoadjuvant therapy with camrelizumab plus FOLFOX without confirmed progressive disease. Of 13 evaluable patients, 1 (8%) achieved pathological complete response (pCR), 3 (23%) had tumor regression grade (TRG) 1, and 10 (77%) showed stage reduction, and eight experienced lymphonodus pCR. The most common grade 3–4 TRAEs included neutropenia (19%), leukopenia (13%), and anorexia (6%) (Liu et al., 2020). The single-arm, phase 2 PANDA (NCT03448835) study, presented at ASCO 2022, demonstrated a 45% pCR and 70.0% major pathologic response (MPR) rate with neoadjuvant atezolizumab plus DOC (docetaxel, oxaliplatin, and capecitabine) treatment in 20 patients. Notably, intestinal-type Lauren classification patients had a 60% (9/15) pCR and 80% (12/15) MPR rate. The median follow-up of 29 months revealed a DFS rate of 75%. Two patients (10%) experienced grade 3 immune-related adverse events (IRAEs) (Verschoor et al., 2022). Another single-arm, phase 2 trial (NCT03488667), also reported at ASCO 2022, showed 19% ypCR (tumor regression score, TRS = 0) and 92% pathological response (TRS ≤2) in 26 of 35 patients treated with neoadjuvant mFOLFOX plus pembrolizumab, with grade 3/4 toxicities reported in 21 patients (Sun et al., 2022). These findings showed a promising pathological response rate with acceptable toxicity profiles when combining chemotherapy with ICIs as neoadjuvant therapy for locally advanced G/GEJ cancer.

The DANTE trial (NCT03421288) was a phase 2b study comparing atezolizumab plus FLOT against FLOT alone in operable G/GEJ adenocarcinoma patients. Patients in Arm A received atezolizumab with FLOT for four neoadjuvant and four adjuvant cycles followed by eight cycles of atezolizumab monotherapy, while patients in Arm B received FLOT alone for 4 + 4 cycles. Presented at ASCO 2022, the combination therapy showed improved tumor downstaging and a higher pCR rate (24% vs. 15%), especially in patients with higher PD-L1 expression (33% vs. 12% in CPS ≥10) and MSI-H tumors (63% vs. 27%). This led to the trial’s advancement to phase 3, focusing on patients with high immune responsiveness (MSI-H, PD-L1 CPS≥1, TMB≥10/MB, or EBV+) (Al-Batran et al., 2022; Al-Batran et al., 2023). The single-arm, phase 2 ICONIC trial (NCT03399071), presented at ASCO-GI 2023, evaluated FLOT-A (FLOT with avelumab) in early-stage operable esophagogastric adenocarcinoma patients with ≥cT2-4 or N+. The trial closed early with a 15% pCR rate in 34 patients, below the 25% target. However, higher PD-L1 CPS was associated with improved TRG3 and reduced TRG4/5 rates, even after excluding patients with dMMR/MSI-H tumors. With a 15.8-month median follow-up, the 12-month PFS was 93.1%, showing promise compared to historical perioperative FLOT results (Gordon et al., 2023).

The phase 2, open-label, randomized NCT04250948 trial evaluated the efficacy of combining toripalimab to perioperative SOX/XELOX in resectable cT3-4 aN + M0 G/GEJ cancer. 108 patients were randomized evenly into either receiving three preoperative and five postoperative cycles of SOX/XELOX (C arm) or receiving toripalimab with SOX/XELOX followed by 6 months of toripalimab maintenance therapy (C + T arm). Results presented at ASCO 2023 revealed a significant increase in the TRG0/1 rate in the C + T arm by 24.0% (*p* = 0.009), with a rate of 44.4% (24/54; 95% CI 30.9%–58.6%) compared to 20.4% (11/54; 95% CI 10.6%–33.5%) in the C arm. Moreover, the C + T arm showed a higher pCR rate of 24.1% (13/54; 95% CI 13.5%–37.6%), which was significantly higher (*p* = 0.039) compared to 9.3% (5/54; 95% CI 3.1%–20.3%) in the C arm. Surgical morbidity (11.8% in the C + T arm vs. 13.5% in the C arm) and mortality (1.9% vs. 0%) and grade 3–4 TRAEs (27.8% vs. 25.9%) were similar between two arms (Yuan et al., 2023). This study provides compelling evidence supporting the combination therapy in the perioperative setting and long-term survival data are anticipated to confirm its survival benefit.

Impressive results were reported in the perioperative and advanced-stage treatment of gastric cancer (GC) with the use of FOLFIRINOX (fluorouracil, leucovorin, oxaliplatin, and irinotecan) (Catenacci et al., 2020; Park et al., 2020). The phase 2 NCT04908566 trial compared toripalimab with FOLFIRINOX (Group A) and toripalimab with SOX (Group B) in the perioperative setting for operable G/GEJ adenocarcinoma. As shown at ASCO 2023, the study enrolled 54 eligible patients (A group 21, B group 33) and achieved R0 resection in all 32 patients who underwent surgery. While the TRG 0-1 rate was higher in Group B, but the difference was not statistically significantly (31.58% vs. 23.08%, *p* = 0.703). PCR was achieved by 15.4% in A and 10.5% in B (2 patients each), with tumor downstaging observed in 71.9% patients (8 in A and 15 in B). TRAEs occurred in 46.3% (25/54) of patients, with 18.5% (10/54) experiencing grade ≥3 TRAEs, including neutropenia, thrombocytopenia, and myelosuppression (Liu et al., 2023). The trial suggests a potential treatment option, yet further investigation is needed to determine the suitability of intensive *versus* simplified treatment for neoadjuvant therapy.

The global phase 3 KEYNOTE-585 trial (NCT03221426) enrolled patients with stage II, III, or IVa G/GEJ cancer. Participants were randomly assigned to either pembrolizumab plus FP/XP (Xeloda and cisplatin) or placebo plus chemotherapy (1:1 ratio, the main cohort). After three cycles of neoadjuvant therapy and subsequent curative surgery, those achieving R0 resection received 14 cycles of adjuvant therapy (3 cycles of combination therapy followed by 11 cycles of pembrolizumab or placebo monotherapy). Additionally, a safety FLOT cohort was introduced based on the AIO-FLOT4 study results, assigning patients randomly to either pembrolizumab or placebo plus FLOT. The primary endpoints were OS, EFS, and pCR. The results, presented at the ESMO Congress 2023, highlighted a notable increase in pCR rates within the main cohort, with a 10.9% improvement (95% CI 7.5–14.8; *p* < 0.00001) observed (12.9% with pembrolizumab vs. 2.0% with placebo). Additionally, the main plus FLOT cohort exhibited a 10.6% increase (95% CI 7.4–14.0; *p* < 0.0001) in pCR rates (13.0% vs. 2.4%). However, the improvement in pCR did not translate into a substantial extension in EFS for either the main cohort (median: 44.4 months vs. 25.3 months; HR 0.81; 95% CI 0.67–0.99; *p* = 0.0198) or the main plus FLOT cohort (median: 45.8 months vs. 25.7 months; HR 0.81; 95% CI 0.68–0.97). Moreover, there was also no significant improvement in OS in the main cohort (median: 60.7 months vs. 58.0 months; HR 0.90; 95% CI: 0.73–1.12). Rates of grade ≥3 drug-related adverse events (AEs) were comparable between the two groups in the main cohort (65% vs. 63%) (Shitara et al., 2023).

The preliminary results of the phase 3, double-blind, randomized MATTERHORN trial (NCT04592913) was also presented at the ESMO 2023. 948 patients with resectable G/GEJ cancer were randomized in a 1:1 ratio to receive durvalumab or placebo plus FLOT for two cycles of neoadjuvant and two cycles of adjuvant therapy, followed by 10 additional cycles of durvalumab or placebo. There was a significant 12% increase (OR 3.08, 95% CI 2.03–4.67; *p* < 0.00001) in the pCR rate, a secondary endpoint, among durvalumab group (19%) compared with placebo group (7%). Additionally, TRG 0/1 rates were higher in the durvalumab group (27% vs. 14%). Surgery completion rate (87% vs. 84%) and R0 resection rate (86% in each arm) were similar between two groups. Treatment with durvalumab resulted in greater surgical downstaging (23% pT0 and 52% pN0) *versus* placebo (11% pT0 and 36% pN0). The rates of grade 3–4 AEs (69% with durvalumab vs. 68% with placebo), TRAEs (95% vs. 94%) and grade 3–4 TRAEs (58% vs. 56%) were comparable (Janjigian et al., 2023). The primary endpoint of EFS is under investigation. While promising, recommending ICIs in perioperative therapy requires further follow-up data.

As to the lack of prolonged survival in KEYNOTE-585 despite improved pCR, it was pointed out at the meeting that immunotherapy was most effective in PD-L1-positive GC, but the patients in this trial were not selected based on biomarkers. In patients with CPS ≥10, pembrolizumab did exhibit a trend toward improved EFS (HR 0.69, 95% CI 0.48–1.01). Additionally, the majority of patients received cisplatin-based chemotherapy instead of the ESMO-recommended FLOT regimen. Notably, MATTERHORN, which has a similar design to KEYNOTE-585 but uses FLOT as the only chemotherapy regimen, achieved higher pCR rates, even in the control arm. Oxaliplatin may be more effective than cisplatin in the perioperative setting.

HLX10-006-GCneo (NCT04139135) is a phase 3 clinical trial comparing serplulimab plus SOX to placebo plus SOX in the perioperative setting for PD-L1 CPS ≥5 GC patients. Patients receive three cycles of neoadjuvant SOX treatment with serplulimab or placebo. After surgery, the serplulimab plus SOX group continues serplulimab monotherapy for 17 cycles, while the control group uses SOX alone for five cycles. The NICE trial (NCT04744649) is a phase 2, open-label, randomized study that compares toripalimab plus SOX/XELOX to SOX/XELOX alone in the perioperative treatment of resectable G/GEJ cancer (cT3-4aNxM0 or cT2N + M0) with PD-L1 CPS ≥5. Additionally, there are two exploratory groups investigating toripalimab plus SOX/XELOX in EBV + or dMMR/MSI-H patients. Each group receives 4 + 4 cycles of perioperative therapy. The phase 2 study NCT04367025 evaluates the perioperative SOX plus camrelizumab in G/GEJ cancer patients with PD-L1 CPS ≥1. The treatment involves 2-4 cycles of neoadjuvant and 2-4 cycles of adjuvant therapy. The phase 2, open-label MONEO (NCT03979131) trial investigates whether adding avelumab to FLOT chemotherapy improves pCR rate in G/GEJ adenocarcinoma compared to the historical data of chemotherapy alone in the perioperative setting. Patients will receive four cycles of neoadjuvant FLOT plus avelumab and four cycles of adjuvant therapy of the same schema, followed by avelumab maintenance therapy up to 1 year. NCT05101616 is a phase 1/2 randomized controlled trial examining neoadjuvant chemotherapy with/without camrelizumab for locally advanced GC (T3-4aN1-3M0). The chemotherapy regimen includes nab-paclitaxel (nab-PTX), S-1, and oxaliplatin. These trials will offer valuable insights into different combinations of chemotherapy and immunotherapy, contributing valuable data to expand the spectrum of treatment options.

Conventional SOX regimen consists of oral S-1 and intravenous oxaliplatin. The phase 3 TACTIC (NCT05593458) study evaluates whether replacing intravenous oxaliplatin with arterially infused oxaliplatin, combined with oral S-1 and sintilimab, can be a better neoadjuvant option for locally advanced G/GEJ cancer. Patients receive either three cycles of conventional SOX chemotherapy plus sintilimab or arterially infused oxaliplatin plus S-1 and sintilimab. Following radical surgery, they undergo three cycles of adjuvant chemotherapy using conventional SOX regimen plus sintilimab, with S-1 administered until 1 year after surgery.

As previously mentioned, dMMR/MSI-H GC patients exhibit a high sensitivity to immunotherapy but a limited response to chemotherapy (Pietrantonio et al., 2019). The DANTE study revealed a remarkable 63% increase of pCR rate in MSI-H patients with the combination of immunotherapy and chemotherapy (Al-Batran et al., 2022). It represents a key predictive biomarker for ICIs. NCT05994456 is an ongoing single-arm phase 2 trial evaluating toripalimab monotherapy in the perioperative management of locally advanced dMMR/MSI-H G/GEJ adenocarcinoma. Another ongoing randomized phase 2 study, NCT05836584, compares perioperative atezolizumab combined with chemotherapy (FLOT or mFOLFOX or CAPOX) to atezolizumab monotherapy. Their results are awaited and more investigations are required to advance our understanding and optimize the perioperative treatment options for these specific patient populations.

### ICIs in combination with chemoradiation therapy

Neoadjuvant chemoradiotherapy is recommended for locally advanced esophagogastric cancer due to its efficacy in reducing the risk of postoperative recurrence by more comprehensively eradicating micrometastases (Wang et al., 2021; Ajani et al., 2022). Radiotherapy at one site may lead to reduction or disappearance of non-irradiated distant tumors or metastatic lesions, which is known as the abscopal effect (Mole, 1953), as it can activate the host immune response (Postow et al., 2012; Ngwa et al., 2018). In addition, radiotherapy has been shown to alter the tumor immune microenvironment including upregulating the expression levels immune checkpoints (Theelen et al., 2019; McLaughlin et al., 2020). The combination of radiotherapy and immunotherapy can synergistically enhance treatment outcomes (Ngwa et al., 2018; Sato et al., 2020), providing benefits in the perioperative management of locally advanced GC patients (Table 3).

**TABLE 3:** Clinical trials of ICIs plus CRT/targeted therapy or dual ICIs in perioperative setting.

Trial	Patients	Agent (target)	Phase, arm	Study design	Size	pCR (%)	MPR (%)
Neo-PLANET (NCT03631615)	-	Camrelizumab (PD-1)	II, single-arm	Camrelizumab + XELOX + cCRT	36	33.3	44.4
SHARED (ChiCTR1900024428)	-	Sintilimab (PD-1)	II, single-arm	Sintilimab + S-1+nab-PTX + cCRT	34	38.2	79.4
RARE (NCT05941481)	-	Tislelizumab (PD-1)	II, single-arm	Tislelizumab + XELOX + cCRT	21	-	-
GERCOR NEONIPIGA (NCT04006262)	dMMR/MSI-H	Nivolumab + Ipilimumab (PD-1, CTLA-4)	II, single-arm	Nivolumab + Ipilimumab	29	58.6	-
INFINITY (NCT04817826)	dMMR/MSI-H and EBV-	Durvalumab + Tremelimumab (PD-L1, CTLA-4)	II, single-arm, multi-cohort	Durvalumab + Tremelimumab	18	60	80
NCT03950271	HER2+	Camrelizumab (PD-1)	II, single-arm	Camrelizuma + Trastuzumab + CAPOX	16	31.3	56.3
NCT04819971	HER2+	Tislelizumab (PD-1)	II, single-arm	Tislelizumab + Trastuzumab + DOS	7	42.9	57.1
NCT05504720	HER2+	Pembrolizumab (PD-1)	II, single-arm	Pembrolizumab + Trastuzumab + FLOT	30	-	-
NCT05218148	HER2+	Sintilimab (PD-1)	II, two-arm	Sintilimab + Trastuzumab + SOX	22	-	-
				SOX only	22	-	-
NCT03878472	-	Camrelizumab (PD-1)	II, single-arm	Camrelizumab + Apatinib + S-1 ± oxaliplatin	19	15.8	26.3
DRAGON-IV/AHEAD-G208 (NCT04208347)	-	Camrelizumab (PD-1)	III, two-arm	SOXRC	256	18.3	51.5
				SOX only	256	5.0	37.8
TAOS-3B-Trial (NCT05223088)	-	Tislelizumab (PD-1)	II, single-arm	Tislelizumab + Apatinib + SOX	25	24	36
TAOS-3B-Trial-2 (NCT05699655)	-	Tislelizumab (PD-1)	II/III, two-arm	Tislelizumab + Apatinib + SOX	65	-	-
				SOX only	65	-	-

XELOX: Xeloda + Oxaliplatin; CAPOX: Capecitabine + Oxaliplatin; DOS: Docetaxel + Oxaliplatin + S-1; FLOT: Fluorouracil + Leucovorin + Oxaliplatin + Docetaxel; SOX: S-1 + Oxaliplatin; SOXRC: SOX + Apatinib + Camrelizumab; nab-PTX: nab-paclitaxel; cCRT: concurrent Chemoradiotherapy; pCR: pathologic Complete Response; MPR: major pathologic response.

The single-arm, phase 2 Neo-PLANET trial (NCT03631615) explored camrelizumab combined with cCRT in locally advanced G/GEJ adenocarcinoma. 36 patients received preoperative sequential treatment with XELOX, cCRT (capecitabine, 45Gy/25f), XELOX, and concurrent camrelizumab since initiating chemotherapy. Of these, 33 patients (91.7%) underwent surgery with all achieving R0 resection. The pCR (ypT0, primary endpoint) rate reached 33.3% (95% CI: 18.6–51.0), meeting the pre-specified endpoint. Other rates included total pCR (ypT0N0, 33.3%), MPR (44.4%), and R0 resection (91.7%). Additionally, 77.8% (28/36) of patients reached ypN0 status. After a 2-year follow-up, PFS and OS rates were 66.9% and 76.1%, respectively. However, grade 3–4 AEs were observed in 86.1% (31/36) of patients, with the most common being decreased lymphocyte count (75.0%, 27/36) (Tang et al., 2022).

In another phase 2, single-arm trial (SHARED, ChiCTR1900024428), 34 patients with locally advanced G/GEJ cancers received neoadjuvant therapy involving one cycle of sintilimab and chemotherapy (S-1 and nab-PTX), followed by 5 weeks of cCRT (45Gy/25F, nab-PTX) and sintilimab, along with an additional cycle of sintilimab and chemotherapy (Jia et al., 2023). After surgery, three cycles of adjuvant sintilimab and chemotherapy were administered. All patients underwent neoadjuvant therapy and achieved R0 resection. The study met its predefined primary endpoint, with 38.2% (13/34) patients achieving pCR (95% CI 22.2–56.4). In addition, 27 patients (79.4%) had MPR. The median DFS and EFS were 17.0 (95%CI 11.1–20.9) and 21.1 (95%CI 14.7–26.1) months, respectively. The median OS was not reached, with 1-year OS rate observed at 92.6% (95%CI 50.1%–99.5%). During preoperative therapy, 17 (50.0%) patients experienced grade ≥3 AEs, primarily myelosuppression.

The findings of Neo-PLANET and ChiCTR1900024428 highlight the promising efficacy of ICIs combined with cCRT for the perioperative management of locally advanced esophagogastric adenocarcinoma. Furthermore, the ongoing randomized phase 2 NeoRacing (NCT05161572) study is investigating the efficacy and safety of perioperative SOX with the addition of sintilimab, with or without preoperative chemoradiation (S-1 orally, 45Gy/25f), for cT3-4 aN + M0 or cT4bNanyM0 G/GEJ cancer (Zhou et al., 2022). Another ongoing single-arm phase 2 RARE (NCT05941481) study is evaluating neoadjuvant chemo-hypofractionated radiotherapy (XELOX, 30Gy/12f) combined with tislelizumab in cases staged as cT1-2N + M0 or T3-T4aNanyM0.

### Dual ICIs strategy

CTLA-4 signaling is crucial in inhibiting the initiation of T-cell responses, while PD-1 plays a significant role later, dampening T-cell activity within the TME (Ye et al., 2023). Theoretically, combining CTLA-4 and PD-1 inhibitors offers synergistic effects (Buchbinder and Desai, 2016). However, the CHECKMATE-649 trial’s nivolumab plus ipilimumab cohort revealed that this dual ICI approach did not offer survival benefits for advanced GC patients and was halted due to severe AEs. Notably, in MSI-H tumors, the combination therapy did result in longer median OS (HR 0.28; 95%CI 0.08–0.92) and a higher objective response rate (ORR, 70%; 95% CI 35–93) compared to chemotherapy (Shitara et al., 2022). The dual ICIs strategy has been explored in several perioperative setting studies (Table 3).

The single-arm phase 2 GERCOR NEONIPIGA (NCT04006262) study included 32 patients with resectable dMMR/MSI-H G/GEJ adenocarcinoma, comprising nine with cT2-T3N0, 22 with cT2-T3N1, and one incorrectly included with cT3N1M1. Neoadjuvant therapy with nivolumab and ipilimumab was administered, followed by surgery and adjuvant nivolumab therapy. Six patients (19%) experienced grade 3/4 neoadjuvant TRAEs. Three patients (one was M1 at inclusion) did not undergo surgery and achieved a full endoscopic remission evidenced by tumor-absent biopsies and had normal computed tomography scans. All 29 surgical patients achieved R0 resection, with 17 (58.6%; 90% CI 41.8–74.1) reaching pCR (ypT0N0, the primary endpoint) (André et al., 2023). These results suggest that MSI-H patients may avoid surgical treatment through dual ICIs strategy.

At ASCO-GI 2023, the phase 2, multicenter, single-arm, multi-cohort trial INFINITY (NCT04817826) was presented, which investigated the combination of tremelimumab plus durvalumab as neoadjuvant (Cohort 1) or definitive (Cohort 2) treatment for dMMR/MSI-H and EBV- (EBV negative) resectable G/GEJ adenocarcinoma. Cohort one started with 18^−ΔΔCT^2-4Nany patients; however, one patient withdrew consent, and two opted out of surgery after achieving a complete clinical-pathological response. Out of the 15 patients assessed, the pCR and MPR rates were 60% and 80%, respectively. Grade ≥3 immune-related adverse events (IRAEs) were observed in three patients, involving colitis, pneumonitis, and liver toxicity. There were two post-operative deaths not related to the cancer or treatment adverse effects, and no recurrences were reported (Pietrantonio et al., 2023). These outcomes encourage further investigation into the non-surgical management using dual immune checkpoint inhibitors, with results from Cohort two anticipated.

In the phase 2 VESTIGE trial (NCT03443856), researchers investigated the efficacy of adjuvant nivolumab and low-dose ipilimumab therapy (nivo/ipi arm) compared to chemotherapy (chemo arm) in 189 stage Ib-IVa G/GEJ adenocarcinoma patients identified as high risk for recurrence (ypN1-3 and/or R1 status) following neoadjuvant chemotherapy (a fluoropyrimidine-platinum regimen) and D2 lymphadenectomy. Presented at ESMO-WCGIC 2023, the findings at a median follow-up of 11.1 months revealed a median DFS of 11.9 months (95% CI 8.4–16.8) for the nivo/ipi arm, significantly shorter than the 23.3 months (95% CI 11.8-not reached) observed in the chemo arm (HR 1.80, 95% CI 1.09–2.98, *p* = 0.02). Additionally, the median OS for the nivo/ipi arm was 25.1 months (95% CI 18.6– not reached), *versus* not reached for the chemo arm (HR 1.79, 95% CI 0.89–3.59, *p* = 0.1) These results led to a halt in further trial enrollment (Smyth et al., 2023). These outcomes suggest that adjuvant chemotherapy might be the preferable option in the adjuvant setting.

### ICIs in combination with targeted therapy

Approximately 15%–20% of G/GEJ cancers exhibit positivity for human epidermal growth factor receptor 2 (HER2+) (Guan et al., 2023). The pivotal ToGA study highlighted the benefits of adding trastuzumab, an anti-HER2 drug, to chemotherapy, extending OS and increasing ORR from 35% to 47% (Bang et al., 2010). This finding established trastuzumab combined with chemotherapy as the primary treatment approach for patients with HER2+ status. Further advancement came with the KEYNOTE-811 trial, which showed that incorporating pembrolizumab into this first-line regimen for advanced HER2+ GC significantly raised the ORR from 51.9% to 74.4% (Janjigian et al., 2021b). Consequently, the combination of pembrolizumab, trastuzumab, and chemotherapy for first-line treatment received accelerated approval from the Food and Drug Administration granted. The potential of this triplet therapy in neoadjuvant therapy for HER2+ G/GEJ cancer deserves further exploration (Table 3).

In the single-arm, phase 2 NCT03950271 study, 22 patients with resectable HER2+ G/GEJ adenocarcinoma received camrelizumab combined with trastuzumab and CAPOX for neoadjuvant therapy. Of these, 16 patients underwent D2 resection, with 9 (56.3%) achieved MPR, including 5 (31.3%) with pCR (ypT0N0M0), and the ORR was 77.3% (Li et al., 2022). Another similar single-arm, phase 2 trial (NCT04819971) demonstrated a pCR rate of 42.9% (3/7) and an MPR rate of 57.1% (4/7) with the perioperative treatment of tislelizumab, trastuzumab and DOS (Zhao et al., 2023). These trials suggest that the triple combination therapy is promising, with encouraging pCR and an acceptable toxicity profile. The single-arm, phase 2 NCT05504720 study is currently investigating the combination of pembrolizumab, trastuzumab and FLOT in this setting and the phase 2 NCT05218148 study is evaluating SOX with sintilimab and trastuzumab *versus* SOX only.

Tumor angiogenesis is pivotal in cancer development. Anti-angiogenic drugs, which inhibit the pro-angiogenic effects of vascular endothelial growth factor (VEGF) on its receptor (VEGFR-2), not only normalize tumor blood vessels but also alter the tumor’s immune environment, promoting the infiltration of CD8^+^ and CD4^+^ lymphocytes, reversing immune suppression to an inflammatory state (Shrimali et al., 2010; Tian et al., 2017; Ciciola et al., 2020). The addition of anti-angiogenic agents to ICIs and chemotherapy regimens may enhance neoadjuvant therapy efficacy. Apatinib, a highly selective VEGFR-2 inhibitor, has shown to extend OS and is approved for third- or later-line treatment of advanced or metastatic G/GEJ adenocarcinoma (Li et al., 2016; Wang et al., 2021).

The phase 2, single-arm NCT03878472 study evaluated the combination of camrelizumab, apatinib, and S-1 with or without oxaliplatin as neoadjuvant/conversion therapy for cT4a/bN + GC. Tumor downstaging was achieved in 76.0% (19/25) of cases. The pCR and MPR rates were 15.8% (3/19; 95% CI 3.4%–39.6%) and 26.3% (5/19; 95% CI 9.1%–51.2%), respectively. Specifically, among cT4aN + patients, 18.2% (2/11) achieved pCR, and 36.4% (4/11) achieved MPR. Following a median observation period of 26.7 months, 55.6% (5/9) of the patients undergoing radical resection were free of recurrence. Notably, there were no reported complications of grade 3 or higher (Li et al., 2021).

The preliminary findings of the phase 3 DRAGON-IV/AHEAD-G208 trial (NCT04208347) were reported at ESMO 2023. 360 patients with cT3-4N + M0 G/GEJ adenocarcinoma were randomized to receive three cycles of either SOXRC (SOX, apatinib and camrelizumab) or SOX monotherapy as neoadjuvant therapy. After radical surgery, the SOXRC group continued with three cycles of triple combination therapy, as well as maintenance therapy with carrelizumab and apatinib, while the SOX group received three cycles of SOX followed by S-1 maintenance therapy. In the ITT population, the SOXRC group showed a significantly higher pCR rate of 18.3% (95% CI 13.0–24.8) compared to 5.0% (95% CI 2.3–9.3) in the SOX group. The ypT0N0 rate in the SOXRC group was 16.7%, and the MPR rate was 51.1%, higher than the SOX group’s 4.4% and 37.8%, respectively. The pCR rates among patients who underwent surgery were 21.3% for SOXRC and 5.8% for SOX, with R0 resection rates of 98.7% and 94.2%, respectively. Toxicities were manageable and did not affect the feasibility of surgery. The study provides a feasible and safe option for resectable G/GEJ cancer patients (Li et al., 2023). Subsequent follow-up data are anticipated.

At ESMO-IO 2022, the single-arm, phase 2 TAOS-3B-Trial (NCT05223088) reported that SOX combined with tislelizumab and apatinib as neoadjuvant therapy for locally advanced G/GEJ cancer led to a 92% ORR and 100% disease control rate in 25 patients. The R0 resection rate was 100%, with pCR and MPR rates of 24% (6/25) and 36% (9/25), respectively. All patients experienced manageable TRAEs (Chen et al., 2022). Furthermore, the ongoing TAOS-3B-Trial-2 (NCT05699655) is comparing this regimen to SOX alone for neoadjuvant treatment in the same population.

## Conclusion and future perspective

Incorporating immunotherapy into adjuvant treatment has yielded disappointing outcomes for unselected patients, potentially due to the near elimination of immunotherapy-sensitive tumor cells post-surgery. However, its effectiveness in specific populations warrants further exploration. The addition of immunotherapy to neoadjuvant chemotherapy has demonstrated superior pCR rates compared to traditional treatments in multiple studies for resectable locally advanced G/GEJ cancer. Moreover, combining targeted therapy or radiation therapy with immunotherapy in neoadjuvant treatments has shown promising outcomes. Optimizing treatment sequences, dose intensity and schedule of these combinations for better efficacy and less toxicity remains a key focus for future research. However, the direct correlation of pCR to long-term survival benefits is not always clear, as seen in the KEYNOTE-585 trial (Shitara et al., 2023). Many studies are still in the preliminary or phase II stages, highlighting the need for long-term follow-up and larger phase 3 randomized controlled trials. Moreover, the choice of chemotherapy agents, particularly the potential superiority of oxaliplatin-based regimens over cisplatin, is crucial when used with ICIs. In addition, neoadjuvant immunotherapy leverages higher levels of endogenous tumor antigen present in the primary tumor to enhance T cell priming while the primary tumor is in place (Topalian et al., 2020). Whether neoadjuvant immunotherapy is sufficient to be a definitive treatment for certain patients, allowing them to avoid surgery, is worth further investigation.

Future research should also focus on identifying effective predictive biomarkers. The 2023 NCCN guideline version two recommends neoadjuvant or perioperative ICIs for dMMR/MSI-H cT2+Nany GC patients. Research shows that GC with high PD-L1 expression, EBV+, and TMB-high responds better to immunotherapy, while *H. pylori* infection may impair its efficacy (Lin et al., 2020; Oster et al., 2022). Circulating tumor DNA, monitored by liquid biopsies, shows potential in predicting ICI responses (Jin et al., 2020; Ma et al., 2023). These biomarkers are worth further investigation and combinatorial biomarker strategies are more reasonable than traditional single immune-specific markers. Additionally, components of the TME such as the extracellular matrix, immune cells, stromal cells, aberrant blood vessels, cytokines, and growth factors play critical roles in tumor growth, development, progression, and treatment response (Mou et al., 2023; Roy and George, 2023; Wong et al., 2023). Multi-omics tools not only facilitate new biomarker discovery but also allow in-depth exploration within TME, including immune cell types, quantities, spatial distribution and various molecules, which could drive advancements of precision immunotherapy (Han and Zhan, 2022).

In conclusion, immunotherapy shows great promise in the management of resectable locally advanced G/GEJ cancer. Effective ICI combinations and further high-quality evidence are needed to firmly establish its role in clinical guidelines. And in-depth research is required to refine patient selection through biomarker optimization.
